# The Function of Necroptosis and Its Treatment Target in IBD

**DOI:** 10.1155/2024/7275309

**Published:** 2024-07-31

**Authors:** Francis Atim Akanyibah, Yi Zhu, Tao Jin, Dickson Kofi Wiredu Ocansey, Fei Mao, Wei Qiu

**Affiliations:** ^1^ Key Laboratory of Medical Science and Laboratory Medicine of Jiangsu Province Department of Laboratory Medicine School of Medicine Jiangsu University, Zhenjiang 212013, Jiangsu, China; ^2^ The People's Hospital of Danyang Affiliated Danyang Hospital of Nantong University, Zhenjiang 212300, Jiangsu, China; ^3^ Department of Gastrointestinal and Endoscopy The Affiliated Yixing Hospital of Jiangsu University, Yixing, China; ^4^ Directorate of University Health Services University of Cape Coast, Cape Coast CC0959347, Ghana; ^5^ Nanjing Jiangning Hospital, Nanjing 211100, Jiangsu, China

## Abstract

Inflammatory bowel disease (IBD), which encompasses Crohn's disease (CD) and ulcerative colitis (UC), is a complicated illness whose exact cause is yet unknown. Necroptosis is associated with IBD pathogenesis, leading to intestinal barrier abnormalities and uncontrolled inflammation. Molecules involved in necroptosis, however, exhibit different expression levels in IBD and its associated colorectal cancer. Multiple studies have shown that inhibiting these molecules alleviates necroptosis-induced IBD. Moreover, due to the severe scarcity of clinical medications for treating IBD caused by necroptosis, we review the various functions of crucial necroptosis molecules in IBD, the stimuli regulating necroptosis, and the current emerging therapeutic strategies for treating IBD-associated necroptosis. Eventually, understanding the pathogenesis of necroptosis in IBD will enable the development of additional therapeutic approaches for the illness.

## 1. Introduction

Inflammatory bowel disease (IBD), which encompasses Crohn's disease (CD) and ulcerative colitis (UC), is a recurrent, persistent inflammatory disorder linked to a higher risk of colorectal cancer (CRC) [1]. IBD can cause abdominal pain, diarrhea, and anemia [2], as well as weight loss [3]. Since 1990, the incidence of IBD has increased in newly industrialized South American, Asian, and African countries [4]. IBD is caused by a combination of genetic susceptibility, stresses in the environment, and widely abnormal intestinal immune reactions to the commensal intestinal microbiota [5]. The causes or risk factors contributing to IBD onset have been summarized in Table 1. IBDs lead to higher rates of intestinal epithelial cell (IEC) death, causing a deterioration of the intestinal barrier, activation of immunological cells, and increased IEC mortality [20].

Necroptosis is a recently identified type of regulated cell death, and additional study is necessary to understand its function in IBD [21]. In IBD and experimental colitis, excessive necroptosis is a key contributor to intestinal barrier abnormalities and uncontrolled inflammation [22]. The receptor-interacting protein kinases (RIPK) 1 and 3, which group together to form necrosomes in the cytoplasm, and the mixed lineage kinase domain-like (MLKL), the final effector pseudokinase, make up the pathway's fundamental machinery [23]. It is widely known that RIPK3-induced necroptosis activates MLKL, increases cytokine expression and alarmins (IL-8, IL-1, IL-33, and high mobility group box 1 (HMGB1)), the nuclear factor kappa-light-chain enhancer of activated B cell (NF-kB) p65 translocation, and the assembly of the NLR family pyrin domain containing 3 (NLRP3) inflammasome [24]. Many inflammatory disorders have signs of a dysfunctional necroptosis process; however, their function in IBD and the fundamental mechanisms remain unknown [22].

The impact of necroptosis on cancer is unclear, as cancerous cells may avoid necroptosis to thrive, indicating the downregulation of critical necroptotic pathway regulators; nevertheless, the expression of important mediators is increased in some cancer forms [25]. There is also a significant shortage of clinical medications that can effectively treat necroptosis in IBD [26]. Hence, we have reviewed the functions of crucial necroptosis molecules in IBD, other stimuli regulating necroptosis, and emerging therapeutic strategies for treating IBD-associated necroptosis.

## 2. Necroptosis Pathways

Necroptosis is carried out by the RIPK1–RIPK3–MLKL signaling cascade, whose dysregulation can result in several human illnesses, including cancer [27]. It is noteworthy that the canonical and noncanonical pathways are the main necroptosis pathways, which have been highlighted below.

### 2.1. Canonical Pathway of Necroptosis

**Figure 1:**
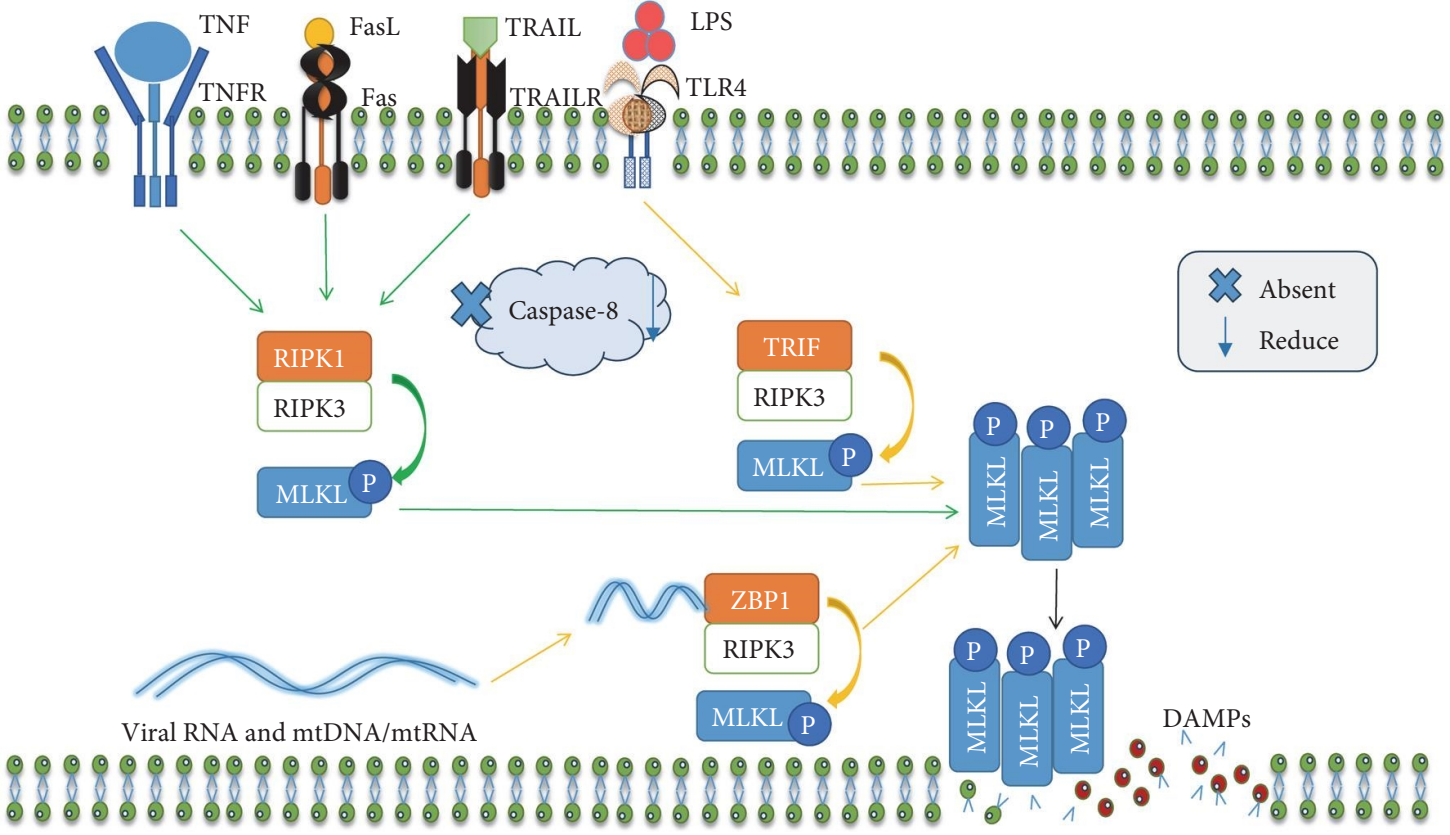
The mechanism of necroptosis. FasL, TRAIL, and TNF start necroptosis by encouraging the development of necrosome complexes. LPS triggers necroptosis through necrosome complex formation mediated by TRIF. ZBP1-bound viral RNA and cellular mtDNA and mtRNA produce RIPK1-independent cell death via the ZBP1–RIPK3 combination. Canonical pathways are highlighted with green arrows, and noncanonical pathways are highlighted in orange.

Necroptosis is initiated by ligand coupling to tumor necrosis factor (TNF) family death domain receptors, pattern-recognizing receptors, and virus sensors. These receptor systems share the ability to attract and activate RIPK3 via proteins that possess the RIPK homology interaction motif (RHIM). RIPK3 recruits and phosphorylates the necroptotic pathway's executor, the pseudokinase MLKL [28], resulting in swelling of the cell, plasma membrane rupture, intracellular material loss, and eventually cell death and inflammation promotion [21]. The RIPK1 kinase and TIR domain-containing adapter-inducing interferon-*β* (TRIF), which regulate cell death and survival, are the initiators of the TNF family members and Toll-like receptor 3/4 (TLR3/4) [28] (Figure 1). Therefore, RIPK1 and RIPK3 are necessary for TNF-induced necroptosis to begin [29]. Interestingly, activation or an increase in RIPK3 and MLKL causes necroptosis when caspase-8 is absent or reduced [30, 31]. This shows the crucial role of caspase-8 in necroptosis. Stimuli such as the activation of death receptors (tumor necrosis factor receptor (TNFR), Fas, and TNF-related apoptosis-inducing ligand receptor (TRAILR)) [32], obatoclax (GX15-070) [33], shikonin [34], vaccinia virus infection [35], and respiratory syncytial virus infection [36] result in necroptosis. It is interesting to note that there is an interaction between necroptosis and apoptosis regarding their death receptors. For instance, TNFR1 [37], Fas [38, 39], and the TNF-related apoptosis-inducing ligand (TRAIL) receptors DR4 [40, 41, 42] and DR5 [41, 42, 43] are additional proapoptotic death receptors. Death receptors can attach to their appropriate ligands and trigger a series of events that can either lead to gene expression or cell death [44]. Therefore, apoptosis signaling by death receptors requires the recruitment of adaptor proteins (TNF receptor-associated death domain protein (TRADD) and Fas-associated death domain protein (FADD)) and caspase-8 and caspase-10, and both caspases may play equivalent roles in apoptosis onset [45, 46, 47, 48]. This may imply that although necroptosis does not require caspase, apoptosis does. Eventually, this pathway has been shown to involve these key structural molecules, RIPK1, RIPK3, and MLKL, when caspase-8 is absent or suppressed.

### 2.2. Noncanonical Pathway of Necroptosis

In contrast to the canonical pathway, which involves the key structural molecules RIPK1, RIPK3, and MLKL to initiate necroptosis in the absence of caspase-8, the noncanonical pathway can induce necroptosis without the presence of all three crucial molecules. For instance, RIPK3-dependent necroptosis is caused by a cytomegalovirus viral infection [49]. Similarly, Z-RNAs produced by replicating influenza A virus activate Z-DNA-binding protein 1 (ZBP1), which then triggers RIPK3-mediated MLKL stimulation in the nucleus, causing necroptosis [50]. These indicate that some viral infections may be dependent on the sensor ZBP1/DAI. Through a RIP homotypic interaction motif-dependent connection of TRIF with RIPK3 kinase (also known as RIPK3), TLR3 or TLR4 directly induces programmed necrosis [51]. While RIPK1 and its kinase activity are not necessary for this pathway to function, MLKL, which is located downstream of the RIPK3 kinase, is still necessary [51] (as shown in Figure 1).

## 3. Key Features of Necroptosis, Apoptosis, and Other Forms of Cell Death

A basic physiological process in all living things is cell death, and various forms of programmed cell death (PCD) have been found [52] involving necroptosis and apoptosis [53]. Main cell death mechanisms, such as necroptosis and apoptosis, usually elicit conflicting immunological responses [54]. Apoptotic death typically results in immune responses that are quiet, while necroptotic death produces chemicals that trigger inflammation, known as necroinflammation [54]. Necroptosis is facilitated by RIPK1, RIPK3, and MLKL, leading to cell enlargement, plasma membrane rupture, leakage of intracellular contents, and ultimately cell death, along with the induction of inflammation [21]. However, MLKL is exclusively associated with necroptosis [55], implying that MLKL might not be present during apoptosis, although RIPK1 and RIPK3 might be. In contrast to structural changes in necroptosis, apoptosis involves structural changes such as nuclear and cytoplasmic condensation, as well as the fragmentation of the cell into membrane-bound, well-preserved pieces. Apoptotic entities are released from surfaces lined with epithelial cells or are engulfed by other cells, where they experience a sequence of transformations similar to in vitro autolysis inside phagosomes and are quickly broken down by lysosomal enzymes from the cells that ingested them [56]. Also, new research shows that RIPK1 and caspase-8 control how apoptosis, necroptosis, and pyroptosis talk to each other, which changes the type of cell death that happens when cell death signals are activated [57]. Caspase-8 triggers death receptor-induced apoptosis and blocks RIPK3–MLKL-mediated necroptosis [58], implying that while caspase-8 is essential for starting apoptosis, necroptosis can proceed without it. Generally, necroptosis and apoptosis may involve RIPK1 and RIPK3. Nevertheless, MLKL is present only in necroptosis, which leads to membrane rupture and inflammation promotion. This may explain why necroptosis is inflammatory while apoptosis is not. Caspase-8 is involved in apoptosis but not in necroptosis.

In contrast to necroptosis and apoptosis, pyroptosis is an inflammatory kind of PCD initiated by certain inflammasomes, resulting in the breakdown of gasdermin D (GSDMD) and the stimulation of dormant cytokines such as IL-1*β* and IL-18 [59, 60]. This implies that while apoptosis is not inflammatory, pyroptosis and necroptosis could be. The effector of pyroptosis is GSDMD [61], while MKLK is the final effector kinase in necroptosis; however, pores can arise from either pyroptosis or necroptosis [61]. Interestingly, GSDMD deficiency delays the rupture of membranes, leading to a shift in cell death appearance back to apoptosis [62]. In the last stage of pyroptosis, caspase-1 in the canonical route and caspase-4/caspase-5/caspase-11 (caspase-4/caspase-5 in humans and caspase-11 in mice) in the noncanonical pathway must cleave GSDMD at D275 (numbering after human GSDMD) into N- and C-termini [63]. This suggests that apoptosis and pyroptosis may require caspases, but necroptosis does not.

Ferroptosis, a nonapoptotic form of cell death, is iron-dependent and is caused by small molecules or conditions that stop the production of glutathione or glutathione peroxidase 4 (GPX4) [64, 65]. Its physical features include smaller-than-normal mitochondria with condensed densities of the mitochondrial membrane, a decrease in or disappearance of the mitochondrial crista, and a rupture of the outer mitochondrial membrane [66]. Unlike necroptosis, where there is plasma membrane rupture, mitochondrial membrane rupture occurs in ferroptosis. A study by Yi and team revealed that the injection of peiminine (PMI) leads to notable elevations in reactive oxygen species (ROS), malondialdehyde (MDA), and iron levels, a decrease in glutathione (GSH) activity, mitochondrial shrinkage, and a reduction in GPX4. However, ferrostatin-1 was able to prevent all of these effects [67]. This further corroborates the idea that in ferroptosis, GSH and GPX4 decrease with a rise in iron levels. Unlike apoptosis and pyroptosis, which depend on caspase, ferroptosis relies on intracellular iron and differs in structure, biochemistry, and genetics from apoptosis, necrosis, and autophagy [68]. Both necroptosis and ferroptosis are inflammatory processes. Necroptosis may be a significant regulatory factor in the pathophysiology of IBD, which offers a fresh concept and approach for examining the disease's potential treatment target in more detail [21].

## 4. Necroptosis in IBD Pathogenesis

### 4.1. Necroptosis Promotes IBD by Controlling Immune Cells and Impairing Intestinal Barrier Integrity

A recent study indicates that changes in the innate lymphoid cells during IBD can significantly contribute to the progression of the disease [69]. Tissue damage, persistent intestinal inflammation, and ongoing responsiveness to microbial antigens are all sustained by innate cells under the direction of inflammatory T cells [69]. Lee and team discovered that CD4+ T lymphocytes expressed more necroptosis mediators in the colon tissue of the UC patient. It was further found that the RIPK3 inhibitor reduced CD4+ T cell necroptosis and T helper-17 (Th17) cell differentiation [70]. Moreover, colitogenic T cells have been shown to initiate and perpetuate colitis [71]. Therefore, necroptosis of T cells may be involved in the onset of IBD. Also, this suggests that necroptosis could potentially play a significant role in regulating immune cells and initiating inflammation in IBD. In other studies, Lu and team found that RIPK1 inhibitors prevent colitis by reducing the infiltration of immunocytes in the lamina propria. The inhibition of RIPK1 led to a delay in the immunological response by altering T-cell activation and differentiation in immune organs [72]. These findings further indicate that T cells may be the most important immune cells in necroptosis and play a significant role in the progression of IBD.

The preservation of host homeostasis depends critically on the intestinal epithelial barrier. It comprises mucus, tight junctions, and epithelial cells [73]. Occludin, claudins, and zonula occludens are tight junction proteins crucial for maintaining the integrity of the epithelial barrier [74]. IBD is a condition characterized by the loss of the integrity of the intestinal epithelial barrier [75]. Therefore, necroptosis disrupting these tight junction proteins may facilitate IBD pathogenesis. Chu et al. [76] found that the neutrophil extracellular trap (NET) promoted necroptosis of IEC. Surprisingly, pMLKL levels increased significantly, destroying the tight junction proteins claudin-1 and occludin. The pretreatment of Nec-1 significantly reduced the degradation of occludin and claudin-1 caused by NETs [76]. Also, pigs treated with Nec-1 to prevent necroptosis exhibited elevated occludin and claudin-1 protein expression [77]. A study in IBD patients found that necroptosis caused by RIPK3 affects the permeability of membranes by altering E-cadherin, occludin, and zonulin-1 [24]. Hence, necroptosis may impair barrier integrity and facilitate IBD. Therefore, necroptosis can regulate immune cells, disrupt barrier integrity, lead to homeostatic imbalance, and ultimately cause IBD.

### 4.2. Role of Necroptosis Molecules in IBD

#### 4.2.1. RIPK1

The key regulator of signaling pathways causing inflammation and cell death, RIPK1, is an underlying target for treatment in medicine [78]. RIPK1 has been associated with several illnesses, including IBD [79]. A RIPK1 inhibitor has been found to reduce tight junction disruption and mitigate intestinal barrier damage and oxidative stress. Moreover, the discharge of chemokines and adhesion mediators from injured IECs has been blocked by RIPK1 inhibitor treatment [72], implying that the pathogenesis of IBD is greatly aided by the activity of RIPK1. Inhibiting the activity of the RIPK1 kinase has significant anti-inflammatory properties that are highly protective against the development of chronic colitis [80]. This further corroborates other findings that RIPK1 contributes to the pathogenesis of IBD. Additionally, it has been demonstrated that (*S*)-5-benzyl-*N*-(5-methyl-4-oxo-2,3,4,5-tetrahydrobenzo[*b*][1,4]oxazepin-3-yl)-1*H*-1,2,4-triazole-3-carboxamide 5 (GSK2982772) has a strong affinity for RIPK1 and is highly effective at inhibiting a variety of TNF-dependent cellular reactions, thereby lowering the number of cytokines that human UC explants spontaneously produce [81]. Surprisingly, mice deficient in RIPK1 and FADD in IECs showed RIPK3-dependent IEC necroptosis, loss of paneth cells, and localized erosive lesions that are inflammatory to the colon [82], implying that RIPK1 deficiency in IEC-specific cells can lead to RIPK3-dependent necroptosis.

However, it has been demonstrated that recurrent infections and early-onset IBD are associated with total *RIPK1* impairment mediated by uncommon homozygous mutations [78]. Similarly, a more recent study has also revealed that mutations in *RIPK1* are linked with infantile-onset IBD and perianal fistulas [83]. As a result, patients who are extremely young and diagnosed with fistula-in-ano and colitis should be checked for *RIPK1* mutations [83], implying that mutations of *RIPK1* may also lead to the development of IBD. These show the relevance of RIPK1 in perpetuating IBD pathogenesis. Generally, it has been shown that RIPK1 regulates necroptosis-induced IBD, and this can be a target for therapeutic strategies.

#### 4.2.2. RIPK3

Moriwaki et al. [84] demonstrated that RIPK3 regulates a distinct necrosis-independent mechanism of inflammation by controlling the expression of cytokines in dendritic cells (DCs). RIPK3 promotes nuclear RelB-p50 activation and caspase-1-mediated pro-IL-1 processing in DCs, ensuring optimum IL-23 and IL-1 expression [84], implying that RIPK3 may facilitate the pathogenesis of IBD. Similarly, a RIPK3 inhibitor has been shown to mitigate the intensity of experimental colitis and decrease inflammation through the suppression of the inflammatory response and necroptosis [70], indicating that RIPK3 may be involved in the pathogenesis of necroptosis-induced UC. Additionally, in the tissues of UC patients that are inflamed, RIPK3 has been reported to be elevated, suggesting that in UC patients, intestinal inflammation is closely related to necroptosis [85]. Moreover, the severity of UC is strongly correlated with RIPK3 expression in the human colon [86]. The RIPK3 inhibitor GSK872, or RIPK3 knockdown, has been shown to counter the suppressive impact of TNF-*α* on multiplication and the promoting impact of TNF-*α* on apoptosis and necrosis in human IECs [86]. All these pieces of evidence unequivocally demonstrate RIPK3's significant involvement in the pathogenesis of IBD through necroptosis.

#### 4.2.3. MLKL

MLKL deficiency has been found to inhibit the activation of proinflammatory mitogen-activated protein kinases. Moreover, loss of MLKL also prevents dextran sodium sulfate (DSS)-induced colitis [87]. Similarly, MLKL deficiency inhibited ileitis caused by the removal of epithelial caspase-8 but only marginally mitigated ileitis in mice deficient in FADD in IECs [88], implying that MLKL promotes necroptosis or that its deficiency prevents necroptosis. This may imply that MLKL is the final effector kinase receptor responsible for initiating necroptosis, and thus, its deficiency could prevent necroptosis. It is noteworthy that for necroptosis molecules to cause IBD, the necrosome RIPK1–RIPK3–MLKL (pMLKL) has to be formed to initiate damage-associated molecular patterns (DAMPs) and promote inflammation. This further indicates that MLKL is involved in the etiology of necroptosis-induced IBD.

### 4.3. Expression Levels of Necroptosis Molecules in IBD

During necroptosis, certain clinical or experimental features are observed. Human genetics have linked the pathophysiology of inflammatory illnesses to the dysregulation of RIPK1 [89]. Patients with *RIPK1* mutations exhibit clinical features such as recurrent febrile episodes, elevated inflammatory markers, diarrhea, anemia, cachexia, and perianal abscesses [83]. Also, a RIPK3 inhibitor has been found to decrease inflammatory cytokines in UC patients. In experimental colitis, RIPK3 inhibitors decrease inflammatory cytokines and improve weight loss, survival, and colon length [70]. Therefore, RIPK3 may be linked to clinical and experimental features such as increased inflammatory cytokines, weight loss, decreased survival, and colon length. Mice lacking MLKL are protected against inflammation and weight loss and have lower mortality rates [90], implying that MLKL presence may lead to weight loss, inflammation, and mortality. From these findings, some molecular targets of necroptosis may exhibit similar characteristics.

Numerous investigations have revealed RIPK3 and MLKL (pMLKL) expressions involved in the etiopathogenesis of IBD. Hence, it is crucial to identify these expression levels for therapeutic strategies. Intriguingly, investigations have been made into RIPK3's surprising role in boosting malignant proliferation and encouraging the production of proinflammatory mediators [91], implicating that these molecules may have different expression levels in IBD and colitis-associated cancer (CAC) (Table 2).

To begin with, Pierdomenico and colleagues found that RIPK3 and MLKL levels increased in patients with inflamed tissues in IBD and allergic colitis (AC). Concurrently, caspase-8 levels decreased, and similar results were observed in vitro [30]. It has been found that patients with CD have higher levels of necroptosis in the distal ileum, which is associated with increased nuclear DAMP release [95], and this further corroborates the findings that CD patients have higher necroptosis molecule expressions. Moreover, it has been found that necroptosis caused by RIPK3 activates MLKL (pMLKL increase) and augments cytokine and alarmin expression, NF-kBp65 translocation, and NLRP3 inflammasome assembly in IBD [24]. Furthermore, RIPK3 is overexpressed in inflammatory tissues in IBD patients compared to controls [92]. The marker's high expression has been found to have a 92.9% accuracy in predicting the existence of IBD [92].

Even though in vitro research indicates that invasive cancer cells lack RIPK3 expression, the biological function of RIPK3 in a situation pertinent to the disease is still unknown [93]. Liu et al. [91] found that both mouse CAC and human colon carcinoma have increased RIPK3 expression, whereas animals lacking in RIPK3 demonstrate considerably reduced colitis-associated carcinogenesis. Nonetheless, mice deficient in RIPK3 are extremely susceptible to CA-CRC and exhibit increased production of tumor-promoting agents and proinflammatory mediators [93]. Additionally, RIPK3 expression is decreased in malignancies in IBD patients, which subsequently demonstrates that RIPK3 expression is downregulated in human CRC and is associated with the development of cancer [93]. This further corroborates other findings by Feng et al. [94], who found that RIPK3 expression is considerably lower in CRC and associated with the primary tumor (T stage), the presence of distant metastasis (M stage), and the American Joint Committee on Cancer (AJCC stage) of CRC.

## 5. Role of Diet and Metabolites, Enzymes, and Immune Cells in Regulating Necroptosis in Intestinal Injury or IBD

**Figure 2:**
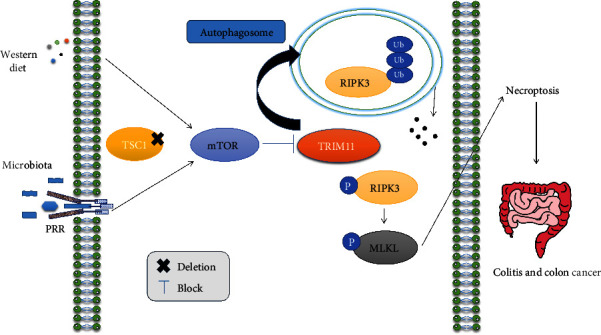
mTOR/RIPK3/necroptosis axis. The microbiota and Western diet play significant roles in promoting intestinal inflammation and cancer through the process of necroptosis.

The major products of the microbial digestion of nondigestible dietary carbohydrates are short-chain fatty acids (SCFAs) and gases [96]. SCFAs include acetate, propionate, and butyrate [97]. It is clear from the research that has been published for more than 30 years that butyrate is crucial to maintaining intestinal homeostasis [98]. Intriguingly, butyrate has been found to cause developmental stage-dependent intestinal damage similar to necrotizing enterocolitis (NEC) [99]. Necroptosis is a key method through which butyrate damages cells in NEC, and necroptosis inhibitors have been proven effective at considerably reducing the enteral toxicity of butyrate [99]. Moreover, the intestinal microbiota's composition and metabolism are significantly influenced by diet, and the primary dietary macronutrients (carbohydrates, proteins, and fats) in terms of quantity, kind, and balance have a significant impact on the large intestine microbiota [96]. It is well documented that eukaryotic cell development and metabolism are coordinated by the mechanistic target of rapamycin (mTOR) with environmental inputs such as nutrition and growth stimuli [100]. Surprisingly, western diet-induced mTOR hyperactivation and *Tuberous sclerosis complex 1* (*Tsc1*) deletion result in epithelial necroptosis by stopping the autophagosome synthesis and TRIM11 action of keeping RIPK3 minimal in IEC and raising RIPK3 levels, leading to barrier disruption and a propensity for DSS-induced colitis and inflammation-related colon cancer [101]. mTOR mainly targets RIPK3 to enhance necroptosis induced by TNF and microbial pathogen-associated molecular patterns (PAMPs) [101] (Figure 2). Also, high-salt diets worsen IBD by activating necroptosis, suggesting new treatments and promising targets for IBD treatment [102]. Besides, the study found that colonic epithelial cells cultured in high salt and sucrose conditions exhibit accelerated RIPK3-dependent necroptosis [102]. These findings support the widely accepted idea that gut microbiota, genetic susceptibility, and environmental and lifestyle factors may contribute to the development of IBD, potentially by regulating necroptosis.

It has been found that the loss of certain enzymes can initiate the release of certain viruses, causing an impaired gut barrier. The SET domain bifurcated histone lysine methyltransferase 1 (SETDB1) is crucial in intestinal epithelial homeostasis [103]. It is noteworthy that SETDB1 loss in intestinal stem cells releases endogenous retroviruses from repression, and the motivated endogenous retroviruses boost viral mimicry, resulting in necroptosis that is ZBP1-dependent, causing intestinal inflammation and irreparably disrupting the epithelial barrier's homeostasis [104]. Necroptosis may also be affected by an enzyme's catalytic subunits. For instance, protein phosphatase 6 (PP6) functions in the cell cycle, DNA injury repair, inflammatory signaling, lymphocyte maturation, virus infection, tumor development or spread, cell or tissue size, and noncoding RNA-mediated regulation, according to mounting evidence [105]. However, genome-wide clustered regularly interspaced short palindromic repeats (CRISPR)/CRISPR-associated (Cas) 9 library testing has revealed that protein phosphatase 6 catalytic subunit (PPP6C) encourages TNF-induced necroptosis [106], implying that PPP6C regulates necroptosis in cecum injury through the TNF pathway. Moreover, the polycomb repressive complex's catalytic component, enhancer of zeste homolog 2 (EZH2), is essential for preserving the integrity of the epithelial cell barrier and homeostasis when there is inflammation. In line with the patients' lower levels of EZH2 expression, the inactivation of EZH2 in IECs sensitizes mice to experimental colitis caused by DSS and TNBS [107]. In the necroptosis model, the necroptotic markers RIPK1 and RIPK3 upregulate concurrently with the downregulation of EZH2. This suggests that EZH2 is crucial for the occurrence of intestinal necroptosis and inflammation [108]. Interestingly, SETDB1 and EZH2 are encoded by the genes *SETDB1* and *EZH2*, respectively. This suggests that genetic factors may play a role in necroptosis, leading to IBD.

IBD and CRC have both been linked to caspase malfunction and intestinal disorders [109]. In patients with CD, caspase-8 seems to be involved in mucosal inflammation and the regulation of paneth cell necroptosis, as well as the potential death of IECs. Therefore, caspase-8 regulators may be employed to prevent cell death in IBD patients [109]. The main mechanism via which necroptosis begins is the release of RIPK3 from caspase-8 inhibition [30], which shows how significant caspase-8 is in regulating necroptosis. The increase or activation of caspase-8 resolves necroptosis, and the reduction, absence, or inactivation of caspase-8 results in necroptosis. In vivo, activation of RIPK3- and MLKL-dependent necroptosis happens when caspase-8 or its adapter, FADD, is absent [31]. According to Pierdomenico et al. [30], caspase-8 reduces, whereas RIPK3 and MLKL increase in the inflamed tissues of IBD and AC patients. This implies that necroptosis only occurs in the absence or reduction of caspase-8. It has also been shown that in the adult mouse small intestine, abnormally regulated microbial and death receptor signaling culminates in the release of MLKL-dependent necroptotic bleeding following endothelial caspase-8 loss, and this confirms that caspase-8 plays a crucial function in protecting gut vascular integrity against microbial commensals [110]. Besides, TLRs are exhibited by IECs, which aid in the identification of microorganisms. The activation of TLR ligands causes a momentary rise in the shedding of epithelial cells, a framework that aids in the clearance of intracellular pathogens and supports the epithelium's antibacterial and antiviral host defense [111]. In mice with caspase-8, TLR activation specifically removed from epithelial cells causes RIPK3-dependent epithelial necroptosis instead of apoptosis, and this shows that caspase-8 is crucial for maintaining the intestinal barrier [111]. Moreover, cellular FLICE-like inhibitory protein (cFlip) regulates the activation of caspase-8 to help IECs survive; hence, adult mice who lack cFlip in their intestinal epithelium pass away from significant tissue loss, epithelial cell death, and intestinal inflammation [112]. These further indicate the role of caspase-8 in causing intestinal death.

Dysfunction of the intestinal immune system is still a principal cause of IBD [113]. IBD's immunological features result from aberrant innate and adaptive immune system reactions [114]. Elshal et al. [115] found that IBD is linked to a disruption in the CD200R1/CD200 axis, where a reduction in DCs that express CD200R1 might play a role in the imbalance between Th17 and regulatory T cells (Treg cells), contributing to the development of IBD. It is interesting to note that other immune cells have been found to affect necroptosis-induced IBD. For instance, macrophages with reduced T-cell immunoglobulin domain and mucin domain-3 (Tim-3) levels produce chemokines that draw neutrophils and release TNF-*α* to trigger neutrophil necroptosis [116]. However, macrophages with Tim-3 suppress the TLR4/NF-*κ*B signaling pathway to prevent neutrophil necroptosis [116]. These results offer fresh perspectives on the pathological process of UC and the role of Tim-3 in modulating neutrophil and macrophage interactions [116]. Also, necroptosis may result from FasL on the surface of lymphocytes [117]. The possibility exists that necroptotic and apoptotic signals are transduced independently, enabling FasL+ lymphocytes to kill tumor cells that evade apoptosis by inducing necroptosis [117]. This may be helpful for IBD complications such as CRC.

It is widely demonstrated that genetic factors, gut microbiota, environmental lifestyle, and immune cells can contribute to the occurrence of necroptosis, ultimately leading to IBD. This statement solidifies the widespread understanding that IBD could be influenced by genetic predisposition, environmental and lifestyle factors, gut microbiota, and immune responses. Hence, necroptosis may be influenced by these factors, which may lead to IBD. Necroptosis may be a crucial regulatory factor in the development of IBD [21]. Therefore, targeting necroptosis molecules may help attenuate IBD.

## 6. Therapeutic Strategies for Regulating Necroptosis in IBD

Several compounds, formulations, extracts, and signaling receptors have been investigated to target specific necroptosis molecules and alleviate IBD caused by necroptosis. Other compounds lack a clear target yet are nonetheless able to lower the expression of RIPK1, RIPK3, and MLKL (Table 3). These, together, help provide promising therapies for treating necroptosis-related IBD.

### 6.1. Compounds Targeting RIPK1 in IBD

RIPK1 inhibitors are being researched as potential treatments for a variety of human disorders, including UC [119] and other gastrointestinal conditions. This will help prevent necroptosis-induced IBD. Necrostatins have been established as RIPK1 kinase first-in-class inhibitors, the primary upstream kinase responsible for necroptosis stimulation [136]. Hence, the administration of necrostatins could help prevent other association molecules from forming to prevent necroptosis. According to Liu et al. [137], the administration of necrostatin-1 inhibits the increase in expression of RIPK1 and RIPK3 and enhances caspase-8 expression in colitis caused by DSS. Additionally, in vitro tests verified necrostatin-1's anti-inflammatory effects, implying that necrostatin-1 may represent a potentially effective therapeutic alternative for the treatment of CA-CRC in IBD patients [137]. In a related investigation, Zhou et al. [22] found that Nec-1s prevent necroptosis, which greatly reduces cell death and alleviates colitis caused by DSS in vivo. Similar findings have also shown that pretreatment of lipopolysaccharide (LPS)-induced intestinal injury by nec-1 reduces RIPK1, RIPK3, and phosphorylated MLKL protein expression [77]. This shows how nec-1 has been extensively used in studies to prevent necroptosis and its potential as a therapeutic strategy in necroptosis-induced IBD and related complications [137]. Interestingly, blocking RIPK1 by Nec-1s in vivo and in vitro dramatically alleviates colitis and cell death, which share the same phenotype with ABIN1 overexpression; hence, ABIN1 activation may be considered a therapeutic strategy for UC [138].

Besides, it has been found that HtrA2 relocates from the mitochondria to the cytosol in response to necroptotic stimulation, interacts directly with RIPK1, and encourages its breakdown during a certain necroptosis time phase [90]. Therefore, the role of UCF-101 (a selective serine protease inhibitor of HtrA2) in reducing RIPK1 phosphorylation and further phosphorylation of RIPK3 and MLKL [90] emphasizes the role of UFC-101 in targeting RIPK1 to prevent necroptosis. Additionally, LY3009120 inhibits necroptosis-related RIPK1 phosphorylation, which leads to RIPK3 and MLKL phosphorylation. LY3009120 suppresses IECs' tendency to necrotize, prevents the loss of intestinal barrier function, and lessens colonic inflammation [26]. These results indicate that LY3009120 is an inhibitor of necroptosis and may be employed as a possible colitis therapy [26]. Moreover, it is widely known that TAK-632 (5), when used in the past, effectively inhibited necroptosis by dual-targeting RIPK1 and RIPK3. However, TAK-632 (25) has been found to selectively target RIPK1 more than RIPK3. The TAK-632 (25) compound demonstrates impressive anti-inflammatory management effectiveness in a mouse model of UC induced by DSS, making it a prospective candidate for UC treatment [118]. It has also been discovered that GSK2982772 (compound 5) strongly attaches to RIPK1 with exceptional kinase selectivity and offers outstanding blocking ability against numerous TNF-dependent cellular responses. Notably, it lessens the cytokine production that arises spontaneously from human UC explants (IL-1 and IL-6) [81]. Currently, a new RIPK1 inhibitor, SZ-15, has been developed, produced, and studied. SZ-15 reduces necroptosis in U937 and HT-29 cells with high efficacy at doses of 1 nanomolar (nM) and 10 nM, respectively. At a dose of 10 nM, it prevents RIPK1, RIPK3, and pMLKL proteins [119]. Similarly, the downregulation of proinflammatory cytokine mRNA expression is also observed. Consequently, SZ-15 has a chance of being used to treat UC clinically [119].

### 6.2. Compounds Targeting RIPK3 in IBD

RIPK3 has been well established to play a crucial role in necroptosis-induced IBD; hence, drugs targeting it will be of great significance to necroptosis-induced IBD amelioration. Lee and colleagues found that GSK'872, a RIPK3 inhibitor, reduces proinflammatory cytokines and necroptosis factors in the colon, which prevents colon damage. The colitis drug GSK-872 reduces the severity of the condition, minimizes inflammation, and supports UC medications that target RIPK3 [70]. Not only has GSK'872 been found to be a RIPK3 inhibitor, but curcumin has also been found to target RIPK3 and inhibit it to prevent necroptosis. According to Zhong et al. [120], curcumin directly binds to RIPK3, as suggested by drug affinity responsive target stability (DARTS) analysis. Curcumin inhibits phosphorylated RIPK3 expression in the epithelium of the intestine, reducing the loss of cells in the intestinal epithelium, improving the role of the intestinal tight junction barrier, and lowering intestinal localized inflammation [120].

Interestingly, other compounds have been found to target both RIPK1 and RIPK3. For example, cardamonin interacts with RIPK1/RIPK3, according to research done using the cellular thermal shift assay (CETSA), DARTS, and molecular docking. According to Shen et al. [121], oral administration of cardamonin attenuates DSS-induced colitis, characterized by intestinal barrier damage mitigation and necroinflammation suppression, decreases pMLKL, and prevents lactate dehydrogenase (LDH) and HMGB1.

### 6.3. Compounds Targeting MLKL in IBD

Numerous studies have shown that necrosulfonamide (NSA) protects against various inflammatory illnesses by preventing MLKL polymerization [122]. Hence, NSA has been identified as an MLKL inhibitor. Yang et al. [122] found that NSA blocks CD4+/CD8+ T-cell and macrophage buildup brought on by DSS in colon tissue and inhibits pMLKL and N-gasdermin D (N-GSDMD) expression.

Unfortunately, the specific compounds targeting MLKL in IBD are limited. Nevertheless, it has been discovered that TC13172 (compound 15) binds covalently to MLKL's cysteine 86 (Cys-86) using the converting biochemistry to chemistry activity-based protein profiling (BTC-ABPP) method. As a result, it becomes a very powerful MLKL inhibitor [139]. Similarly, recent research has led to the discovery of a novel family of MLKL inhibitors, uracil derivatives. These highly novel, potent compounds 56 and 66 are formed from the xanthine MLKL inhibitor TC13172 through scaffold morphing [140]. These compounds only slightly prevent MLKL oligomerization but hinder the membrane translocation of MLKL substantially [140]. The compounds are appealing starting points for future therapeutic development for MLKL-related illnesses due to their distinct mode of action from TC13172 [140].

### 6.4. Herbal Formulations and Therapeutic Extracts in Regulating Necroptosis

Compared to compounds that target a specific necroptosis molecule to stop and reduce other necroptotic pathways, these extra compounds (derived from extracts) and formulations do not have a target but aid in lowering necroptosis molecule expression. Wu-Mei-Wan (WMW) has been successfully used in clinical treatment for enteritis since its inception in China in ancient times [141]. It has been shown that the necroptotic signal molecule RIPK3's O-linked N-acetylglucosaminylation (O-GlcNAcylation) has a protective effect on gut inflammation. Wu et al. [123] recently found that WMW reduces O-GlcNAcase (OGA) activity while enhancing O-GlcNAc transferase (OGT) activity, raising RIPK3 O-GlcNAcylation, and hindering RIPK3 and MLKL binding, which prevents necroptosis and alleviates colitis in mice.

Lee et al. [125] found that *M. officinalis* bark extract (MBE) decreases the activation of the proinflammatory cytokine IL6, suppresses necroptosis indicator expression (pRIP3 and pMLKL) in mice with colitis brought on by DSS, reduces necroptosis-induced reactive oxygen species (ROS) production, and lowers COX2 expression, a target protein of ROS. Moreover, hesperetin has been demonstrated to greatly lessen the manifestations of DSS-induced colitis, suppress RIPK3 and MLKL expression, and deactivate their necroptosis signaling pathways [126]. In the same way, celastrol administration has been found to relieve the severity of colitis by decreasing IL-1*β*, IL-6, myeloperoxidase (MPO), RIPK3, and MLKL levels while increasing active caspase-8 levels and E-cadherin [128]. Equally important is patchouli alcohol (PA), a major active component in *Pogostemon cablin*, which has been found to suppress DSS-induced cell death signaling by downregulating the necroptosis-related RIPK3 and MLKL proteins, suppressing inflammation, and preserving the intestinal epithelial barrier's integrity [133]. In another study, Zhou et al. [132] showed that M10 can repair intestinal barrier structures that have been harmed by DSS, block the inflammatory colonic epithelium's NF-*κ*B and IL-6 signaling pathways from activating, and finally lower the TNF-*α* pathway, which in turn prevents necroptosis (pRIPK3 and pMLKL) in the inflammatory colonic mucosal cells.

15,16-Dihydrotanshinone Ӏ (DHT) has been investigated to have a protective effect on UC by reducing the expression of RIPK1, RIPK3, and MLKL brought on by DSS [127]. Additionally, DHT suppresses the function of myeloperoxidase (MPO), inducible nitric oxide synthase (iNOS), and cyclooxygenase-2 (COX-2) expression in colon tissues and lowers TNF-*α*, IL-1*β*, IL-6, and HMGB1 serum levels [127]. Similarly, eicosapentaenoic acid (EPA) and docosahexaenoic acid (DHA) have been shown to reduce deoxynivalenol (DON)-induced damage to intestinal porcine epithelial cells by downregulating RIPK1, RIPK3, pMLKL, phosphoglycerate mutase family 5 (PGAM5), dynamin-related protein 1 (Drp1), and HMGB1 protein expressions [131]. Notably, the expression of iNOS, COX-2, RIPK1, RIPK3, MLKL, cytokines, and nitrite produced by LPS and LPS plus Z-VAD is significantly reduced by neferine, a naturally occurring alkaloid, according to Wu et al. [129]. Besides, Alagbaoso et al. [124] found that the polysaccharide extract significantly reduces the amount of pMLKL in the colon of animals with colitis by blocking the necroptosis signaling cascade of RIPK1–RIPK3–MLKL.

Indole-3-carbinol (I3C) is a natural dietary agonist of the aryl hydrocarbon receptor (AHR). I3C inhibits the stimulation of RIPK1 and subsequent necrosome formation, reduces NF-*κ*B stimulation, and reduces colonic inflammation. I3C offers protection in mice with colitis caused by DSS by activating AHR [130].

### 6.5. Targeting Signaling Receptors as Therapeutic Strategies in Necroptosis

**Figure 3:**
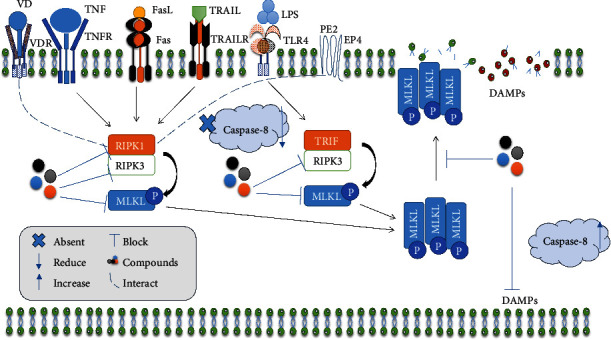
Therapeutic strategies in necroptosis-induced IBD. Compounds and signaling receptors that target RIPK1, RIPK3, and MLKL prevent the release of DAMP, leading to the maintenance of intestinal barrier integrity. Caspase-8 is upregulated/activated during this process.

The possibility that receptors in specific signaling pathways could be used as a treatment target for controlling necroptosis is also intriguing. Patankar et al. [20] found that the IECs' E-type prostanoid receptor 4 (EP4) signaling interacts with RIPK1 to prevent TNF-induced necroptosis. This shows that EP4 enhances colitis resolution by inhibiting IEC necroptosis [20]. Additionally, Shi and colleagues discovered that the intestinal vitamin D signaling pathway inhibits necroptosis. The vitamin D receptor (VDR) moves to the cytosol from the nucleus to stop RIPK1/RIPK3 from forming by binding to RIPK1, and this provides some theoretical support for an innovative approach to treating IBD in clinical settings [142]. Surprisingly, HuanglianGanjiang Tang, a traditional Chinese medicine, has been found to reduce colitis caused by DSS by suppressing colonic necroptosis and increasing VDR levels [134]. Also, an oral EP4-selective agonist, KAG-308, inhibited the development of colitis, thus preventing colorectal carcinogenesis [135]. This drug may have the potential to inhibit necroptosis. Therefore, it could represent a novel therapeutic approach for treating UC [135]. These show the importance of targeting other receptors that may prevent necroptosis-induced IBD for therapeutic purposes. Generally, it has been demonstrated that there are compounds that target necroptosis molecules with specificity using molecular docking, DARTS analysis, CETSA, and other methods. Other compounds lower the expression of these molecules without specificity; hence, these mechanisms may be used as therapeutic approaches to prevent necroptosis-induced IBD and its related complications (Figure 3).

## 7. Conclusion and Perspective

IBD, which encompasses CD and UC, is a complicated illness whose cause is yet unknown. Necroptosis is associated with IBD pathogenesis, leading to intestinal barrier abnormalities and uncontrolled inflammation. It has been discovered that stimuli, including the Western diet, enzymes, immune cells, gut microbiota, and caspase-8, control intestine damage mediated by necroptosis. Molecules involved in necroptosis display varying expression levels in IBD and its related complications, such as CRC. Numerous studies have shown that inhibiting these molecules improves IBD. Also, there is a severe lack of clinical medications for treating IBD caused by necroptosis. Therefore, compounds targeting these molecules have been suggested to have potential clinical importance in the treatment of necroptosis. Thus, in the future, further exploration of specific drugs targeting therapeutic receptors and necroptotic molecules is suggested for usage in clinical settings. Studies on additional pathways to improve the effectiveness of treatments are encouraged.

## Figures and Tables

**Table 1:** The factors associated with the onset and progression of IBD.

Factors	Examples	References
Genetic	Atg16L1 T300A, frameshift mutation in NOD2, CLEC7A rs2078178 AA genotype, IL23R, JAK2, and STAT3 polymorphisms	[6, 7, 8, 9]

Environmental/lifestyle	Consumption of sugary beverages, dietary LCFAs (especially palmitic acid), smoking, no regular involvement in sports during youth and early adulthood, excess weight during childhood, and increased consumption of ultraprocessed food	[10, 11, 12, 13, 14]

Immunological	Innate lymphoid cells, CD27-IgD- B, human B cells express granzyme B (GrB)-expressing CD19 (+) and IgA (+) cells	[15, 16, 17]

Gut microbial	Enterotoxigenic *Bacteroides fragilis*, *Escherichia coli*	[18, 19]

**Table 2:** Role of necroptosis molecule expression in IBD and associated complications.

Model	Type of specimen	Type of condition(s)	Necroptosis molecule(s) expression	Mechanism of action	Reference
Human and in vitro	Biopsy from the ileum and colon and adenocarcinoma cell line HT29	CD and UC	RIPK3 and MLKL increase	IBD and AC patients' inflamed tissues show an increase in RIPK3 and MLKL but a decrease in caspase-8	[30]

Human	Intestinal biopsy	CD and UC	RIPK3 increases	RIPK3 expression levels increase in patients with inflamed tissues	[92]

In vitro	Intestinal cell line HCT116RIP3 and intestinal mucosal biopsies	CD and UC	MLKL (pMLKL) increases (RIPK3-driven)	Elevates IL-8, IL-1*β*, IL-33, and HMGB1, NF-kBp65 translocation, and NALP3 inflammasome assembly. It also influences membrane permeability by changing cell–cell junctional proteins (E-cadherin, occludin, and zonulin-1)	[24]

Animal and human	Colon tissue and CRC patients' tissue	CAC and CRC	RIPK3 increases	RIPK3 boosts the growth of premalignant IECs and stimulates myeloid cell-induced adaptive immune suppression. The development of CAC is aided by JNK and CXCL1 signaling that is induced by RIPK3	[91]

Human and animal	Intestinal sample from surgical specimen and colon sample	IBD-associated CRC	RIPK3 reduces	RIPK3 reduction correlates with cancer development	[93]

Human	CRC and non-CRC tissues and human colon cancer cell line RKO	CRC	RIPK3 reduces	RIPK3 is downregulated in CRC and is linked to the T, M, and AJCC stages of the disease	[94]

**Table 3:** Therapeutic strategies for regulating necroptosis in IBD.

Therapeutic strategies	Model(s)	Target	Mechanism	Reference
Compounds targeting RIPK1				
Necrostatin-1	Pig	RIPK1	Reduces RIPK1, RIPK3, and phosphorylated MLKL protein expression and decreases jejunal macrophages and monocytes, as well as serum and jejunal proinflammatory cytokines	[77]
UCF-101	Mouse and in vitro	RIPK1	Reduces inflammation in the colon, maintains intestinal barrier function, and suppresses pRIPK1 and subsequent pRIPK3 and pMLKL during necroptosis	[90]
LY3009120	In vitro and in vivo	RIPK1	Prevents pRIPK1 and subsequent pRIPK3 and pMLKL, maintains intestinal barrier function, and reduces inflammation in the colon (TNF-*α*, IL-6, and IL-1*β*)	[26]
TAK-632 (25)	In vivo and in vitro	RIPK1	Inhibits phosphorylation of RIPK1, RIPK3, and MLKL, as well as reducing IL-6 and IL-1*β* mRNA expression	[118]
GSK2982772 (compound 5)	Human, mouse, and in vitro	RIPK1	Compound 5 connects to RIPK1 with high kinase selectivity to prevent many TNF-dependent cellular responses and suppress the spontaneous production of cytokines	[81]
SZ-15	In vitro and in vivo	RIPK1	Prevents RIPK1, RIPK3, and pMLKL proteins and downregulates the mRNA expression of proinflammatory cytokines such as TNF-*α*, IL-1*β*, IL-22, and IL-6	[119]
Compounds targeting RIPK3				
GSKʹ872	Mouse, human, and in vitro	RIPK3	Suppresses necroptosis factors (RIPK3 and p-MLKL) and proinflammatory cytokines and prevents colon damage	[70]
Curcumin	In vitro and in vivo	RIPK3	Inhibits the expression of p-RIPK3 and MLKL, reduces IEC loss, improves the function of the intestinal tight junction barrier, and reduces local intestinal inflammation	[120]
Compound targeting RIPK1/3				
Cardamonin	In vitro and in vivo	RIPK1/3	Mitigates intestinal barrier damage, suppresses necroinflammation, reduces pMLKL, and inhibits LDH and HMGB1	[121]
Compound targeting MLKL				
Necrosulfonamide	In vitro and in vivo	MLKL	Suppresses the discharge of inflammatory drivers and LDH and the expressions of pMLKL and N-gasdermin D	[122]
Herbal formulation				
Wu-Mei-Wan (WMW)	Mouse	—	WMW alleviates TNBS-induced colitis in mice by suppressing necroptosis through elevating RIPK3 O-GlcNAcylation	[123]
Natural plant/animal extracts				
Polysaccharides from *Lentinus edodes*	Mouse and in vitro	—	Inhibits the RIPK1–RIPK3–MLKL necroptosis signaling cascade, which results in a reduced level of pMLKL in the colon of mice with colitis and inhibits necroptotic cell death	[124]
*Magnolia officinalis* bark extract (MBE)	In vitro, animal, and in vivo	—	Decreases the activation of the proinflammatory cytokine IL6 and reduces the expression of necroptosis markers, necroptosis-induced reactive oxygen species (ROS) production, and COX2	[125]
Hesperetin	Mouse and in vitro	—	Inhibits the expressions of RIPK3 and MLKL, inactivates RIPK3/MLKL necroptosis signaling, and alleviates TEER reduction	[126]
15,16-Dihydrotanshinone Ӏ (DHT)	Mouse and in vitro	—	DHT slightly reverses the increase in RIPK1, RIPK3, and MLKL expression as well as the decrease in expression of caspase-8 in colon tissues	[127]
Celastrol	Mouse	—	Decreases RIPK3 and MLKL; increases active caspase-8 levels; suppresses IL-1*β*, IL-6, and myeloperoxidase (MPO) levels; and increases E-cadherin levels	[128]
Neferine	Mouse and in vitro	—	Inhibits iNOS, COX-2, RIPK1, RIPK3, MLKL, and cytokines and increases the expression of caspase-8	[129]
Indole-3-carbinol (I3C)	Mouse and in vitro	—	Inhibits the stimulation of RIPK1 and subsequent necrosome formation, reduces NF-*κ*B stimulation, and ameliorates colonic inflammation	[130]
EPA and DHA	In vitro	—	Downregulates TNFR1, RIPK1, RIPK3, pMLKL, phosphoglycerate mutase family 5 (PGAM5), dynamin-related protein 1 (Drp1), and HMGB1	[131]
M10	Mouse	—	Prevents the phosphorylation of RIPK3 and MLKL, as well as the NF-*κ*B and IL-6 signaling pathways	[132]
Patchouli alcohol (PA)	Mouse	—	Suppresses RIPK3 and MLKL proteins and inflammation	[133]
Drugs targeting signaling transduction molecules				
HuanglianGanjiang Tang	Mouse	VDR	Prevent colonic necroptosis by boosting VDR levels	[134]
KAG-308	Mouse	EP4	Prevents the onset of colitis and promotes mucosal repair	[135]

## Abbreviations

AC: Allergic colitis
AJCC stage: American Joint Committee on Cancer
Atg16LI: Autophagy-related 16-like 1
CAC: Colitis-associated cancer
CD: Crohn's disease
COX-2: Cyclooxygenase-2
CRC: Colorectal cancer
CXCL: C-X-C motif chemokine ligand
DAMPs: Damage-associated molecular patterns
DON: Deoxynivalenol
DSS: Dextran sodium sulfate
EP4: E-type prostanoid receptor 4
Fas: Cell-surface Fas receptor
FasL: Fas ligand
HMGB1: High mobility group box1
IEC: Intestinal epithelial cells
Ig: Immunoglobulin
IL: Interleukin
iNOS: Inducible nitric oxide synthase
JAK2: Janus kinase 2
JNK: c-Jun N-terminal kinase
LCFAs: Long-chain fatty acids
LDH: Lactate dehydrogenase
LPS: Lipopolysaccharide
M stage: Presence of distant metastasis
MLKL: Mixed lineage kinase domain-like
MPO: Myeloperoxidase
mt: Mitochondrial
mTOR: Mechanistic target of rapamycin
NF-*κ*B: Nuclear factor kappa-light-chain-enhancer of activated B cells
NOD2: Nucleotide-binding oligomerization domain-containing 2
pMLKL: Phosphorylated mixed lineage kinase domain-like protein
RIPK: Receptor-interacting protein kinase
STAT3: Signal transducer and activator of transcription 3
T stage: Primary tumor
TEER: Transepithelial electric resistance
TLR: Toll-like receptor
TNF: Tumor necrosis factor
TRAILR: TNF-related apoptosis-including ligand receptor
TRIF: TIR domain-containing adapter-inducing interferon-*β*
TRIM11: Tripartite motif containing 11
*TSC1*: *Tuberous sclerosis complex 1*
Ub: Ubiquitin
UC: Ulcerative colitis
VDR: Vitamin D receptor
ZBP1: Z-DNA-binding protein 1.
